# Life Events and the Onset of Celiac Disease from a Patient’s Perspective

**DOI:** 10.3390/nu5093388

**Published:** 2013-08-28

**Authors:** Carolina Ciacci, Monica Siniscalchi, Cristina Bucci, Fabiana Zingone, Ivonne Morra, Paola Iovino

**Affiliations:** Department of Medicine and Surgery, University of Salerno, via S.Allende 84081, Salerno 84081, Italy; E-Mails: monicasiniscalchi@aliceposta.it (M.S.); cristinabucci@hotmail.it (C.B.); z.fabiana@hotmail.it (F.Z.); ivonne.morra@gmail.com (I.M.); piovino@unisa.it (P.I.)

**Keywords:** celiac disease, life events, symptoms, onset of disease, stress, pregnancy

## Abstract

Stressful events have been investigated in various immune-mediated diseases but not in celiac disease. Our aim was to examine the relationship of stressful events assessed by the standardized interview of Paykel with the diagnosis of celiac disease in comparison to patients, with a diagnosis of gastroesophageal reflux disease used as the control group. Adults with celiac disease (*n* = 186) reported more frequent and more severe life events in the years prior to the diagnosis than control patients (*n* = 96) (67.2% *vs.* 37.5%, *p* < 0.001, mean Paykel score 11.5 *vs.* 13.4, *p* = 0.001, respectively). Findings were not significantly different between celiac disease and control patients for the time lapse between the event and the diagnosis (mean 5.5 *vs.* 5.7 months). Pregnancy was defined as a negative event by 20.3% of celiac women, but never by control women. Findings were confirmed when analyses were repeated in the subgroup of patients of both groups with diagnosis made within one year of onset of symptoms. Data indicate that, before diagnosis, the number of stressful events in celiac disease was more frequent although less severe than in the control group suggesting that life events may favor the clinical appearance of celiac disease or accelerate its diagnosis.

## 1. Introduction

There has recently been an increasing awareness of the importance of psychosocial factors on the course of the celiac diseases (CD) [1,2]. Several studies investigated the association between life events and the development of chronic diseases [3], in particular gastrointestinal diseases such as inflammatory bowel disease [4,5,6,7] or in general other chronic diseases such as multiple sclerosis [8], rheumatoid arthritis [9], diabetes [10], skin diseases [11], mania [12], bipolar [13] and affective disorder [14]. Overall, there is an increasing evidence of the role of stressful life events in the onset of immune-mediated diseases [6,15], although the reviews point out the weakness of the majority of the published studies because of methodological issues (study design, controls, scale and timing) and small-sample size. Studies on the relationship between CD and stressful life events are, to our best knowledge, not available. Theoretically, a life event could relate to the onset of CD through at least two mechanisms. Firstly, a stressful life event pushes the person to seek medical consultation, because pre-existing symptoms become more important in their perception. Secondly, affecting the primary mechanism of the disease itself, for instance in the case of CD, through an event inducing psychological modulation of symptoms in a genetically-determined immunological response to gluten. Methodologically standardized questionnaires are considered an adequate tool for investigation about life events when administered by specialized personnel because it is difficult to collect reliable and measurable information about life events and to assess their impact on disease [11]. Conversely, the notable limitation of those studies that explored the role of stressful life events in the onset of immune-mediated diseases, is that the patient may contribute to the misinterpretation of events due to several factors such as the misperception of reality and the altered individual sensitivity that could be features of premorbid personality and/or psychic disturbances. Poor social network and unemployment are additional important factors of vulnerability. Moreover, the impact of life events may be retrospectively the object of interpretation as the patient tries to find an explanation for his/her illness. However, the use of a preformed list of events, of adequate control subjects and, more importantly, limiting the time lapse from the event and the onset of symptoms as much as possible to reduce the recall bias, should guarantee the reliability of the findings.

To explore our hypothesis that there is a possible relationship between life events and the onset of CD, we examined the prevalence of life events prior to the diagnosis in adult CD patients compared to patients with another chronic gastroenterological disease.

## 2. Experimental Section

### 2.1. Patients

#### 2.1.1. CD Group

Patients with newly diagnosed CD and adult age (>18 years) were consecutively recruited in the study at the Gastroenterology Unit, University of Naples “Federico II”, which is a tertiary center for food intolerance and celiac disease. CD was diagnosed based on anti-human tissue transglutaminase and antiendomysium positivity in presence of normal total IgA level and on a positive gluten-related damage at well-performed intestinal biopsies [16,17]. Exclusion criteria were as follows: asymptomatic CD diagnosed in first-degree relatives of a celiac patient; older age (>75 years); signs/symptoms suggesting the onset of CD in pediatric age (history of recurrent gastrointestinal symptoms, reported failure to thrive and/or presence of dental enamel defects or dental hypoplasia); reported use of cannabis or other drugs; alcohol abuse; previous diagnoses of brain disorders (e.g., epilepsy, atassia, *etc.*); previous diagnoses of depression or the actual existence of a depressed mood or of depression as assessed by the modified Zung Depression Rating [18,19].

#### 2.1.2. Control Group

Among gastrointestinal diseases, gastroesophageal reflux disease (GERD) was chosen as the control disease for its similarities to CD in at least two aspects: both of them are chronic diseases and symptoms are subtle in early stages and become progressively more evident over the time up to a climax when often upper endoscopy is performed to obtain a definitive diagnosis [20]. Conversely, GERD is independent from immunological disorders. In fact, the immune-mediated aspects in GERD are usually absent or of minor importance than in CD. Thus, patients with GERD symptoms undergoing upper endoscopy at the Federico II Gastrointestinal Unit that revealed non erosive esophageal disease (NERD) or esophagitis (Los Angeles grade A) were enrolled in this study as a control group. During upper endoscopy, GERD patients underwent jejunal biopsy in order to exclude CD or other causes of malabsorption. Inclusion criteria for GERD were age between 18 to 75 years and negative serum antitransglutaminase antibodies and no IgA deficiency. Exclusion criteria were similar to those for CD patients including signs/symptoms suggesting the onset of GERD in pediatric age; reported use of cannabis or other drugs; alcohol abuse; previous diagnoses of brain disorders (e.g., epilepsy, atassia, *etc.*); previous diagnoses of depression or the actual existence of a depressed mood or of depression as assessed by the modified Zung Depression Rating [18,19].

All patients gave their written informed consent and the study protocol was approved by the Ethic Committee (Diagnosis and Follow-up of Celiac Disease protocol).

### 2.2. Assessment of Symptoms

A previously published questionnaire was used to routinely assess the presence of gastrointestinal symptoms such as abdominal pain, diarrhea, constipation, dyspepsia-like symptoms, GERD symptoms and weight loss [21,22].

### 2.3. Assessment of Life Events

Information about incident events in the last year prior to the diagnosis were collected with the use of “The Interview for Recent Life Events” of Paykel [23,24,25,26] administered by a specialized psychologist (MS) who was unaware of the final diagnosis. The interview categorizes life events of moderate to severe degree in 10 groups as follows: employment, education, financial status, somatic health, loss (death of close relatives), living place, relationship, criminality, family and social problems, marital problems. In the Paykel scale, there are several possibilities to point to a personal disease (admission to the hospital, severe disease, minor disease, pregnancy, abortion, childbirth) although the interview does not allow for specification of the diagnosis or pathological pregnancy. When an event was reported, information was collected also about the date of the event. In the case of multiple reported events, analyses on the date and the quality of the event were based on the event defined as “dominant” by the participant. All events, dependent or independent from illness were recorded. The interview requires approximately 25–70 min, depending on the number of events, the difficulties in obtaining information from subjects and the complexity of the reported event. The Paykel’s interview includes the pregnancy as a possible stressful event. Thus, data were collected also on previous pregnancies. The Paykel’s interview also allows an assessment of the level of independence of the event in a 5-point scale in relation to the illness under consideration and the negative objective impact, and a subjective judgement of the expected stressfulness of the event for an average person, also in a 5-point rating scale. Moreover, the Paykel’s interview attributes an objective normative value for each event (the higher the score, the more severe is an event).

### 2.4. Statistical Analysis

ANOVA were used for analyses on continuous data and reported as means ± standard deviation (SD). Differences in frequencies between groups were calculated with the χ^2^ square test. Odds ratios (OR) ± 95% confidence interval (CI) were used to analyze the risk of having life events in CD compared to the control group. The SPSS software package for Windows [27] was used for statistical analysis.

## 3. Results

Two-hundred and twenty-nine patients newly diagnosed adult patients with celiac disease were eligible. A total of 186 (mean age ± SD: 37.6 ± 12.5 years) fulfilling the inclusion criteria were enrolled in the study, and 43 patients were not included because of exclusion criteria. The majority of CD patients were feminine gender (*n* = 152, 81.7%), with age <40 years (*n* = 117, 63%). Ninety-six GERD patients were selected as the control group. They were similar for gender (women *n* = 70, 72.9%, χ^2^ 1.842, *p* = 0.2) and age (mean age ± SD 36.2 ± 11.8 years, *p* = 0.4) distribution to the CD group. As for the BMI, as expected, it was higher in GERD than in CD patients (mean BMI ± SD: 24.7 ± 3.1 in GERD patients and 22.3 ± 3.8 in CD patients, *p* = 0.000), but none of them showed a BMI greater than 30. None of them refused to answer the questionnaires.

CD and control group did not differ in the length of the period with symptoms prior to diagnosis (4.87 ± 5.8 *vs.* 5.56 ± 3.0 years, *p* = 0.4). Gastrointestinal symptoms (diarrhea and/or abdominal pain) were present in 43% of CD and 22% of control group patients (χ^2^ 11.402, *p* < 0.001). Dyspepsia was present in 41% of CD and 62% of control group patients (χ^2^ 9.956, *p* = 0.002). Weight loss was present in 24% of CD and 12% of control group patients (χ^2^ 5.677, *p* = 0.017). GERD symptoms were present in 46% of CD and 100% of Control group patients (χ^2^ 77.640, *p* < 0.001).

CD patients were statistically more likely to have a life event prior to the diagnosis (OR 3.495% CI 1.766–6.606) compared to patients in the GERD group although the severity of the event assessed by the Paykel’s interview was significantly higher in control group patients than CD (Table 1). In the comparison between the two diseases, differences were not significant in the time lapse between the date of the event and the date of the diagnosis. Due to the higher prevalence of women in both groups a gender-analysis was performed. A significantly higher prevalence of events was observed in women in the CD group than in the control group (71.1% *vs.* 34.3%, χ^2^ 16.727, *p* < 0.001), while men did not differ in the frequency of events between groups (50.0% *vs.* 46.2%, χ^2^ 0.056, *p* = 0.8).

**Table 1:** Reported number of events according to the Paykel’s interview, time lapse from the event to the diagnosis, and normative values (the standardized weight of each event in the scale) in coeliac disease (CD) and control gastroesophageal reflux disease (GERD) subjects.

Variables	CD	GERD	*p*
Number of patients	186	96	
% with events	67.2%	37.5%	<0.001 *
Normative value of event, Paykel score (means ± SD)	11.5 ± 4.8	13.4 ± 4.3	0.001 **
Time lapse between event and diagnosis (months, means ± SD)	5.5 ± 4.1	5.7 ± 6.2	0.9 **

SD = standard deviation; * Chi square test, ** ANOVA.

Pregnancies were reported in 79 CD and in 22 control group women. Number of pregnancies/women was 1.03 ± 1.13 for celiac women and 1.00 ± 0.9 for disease control women (*p* = 0.9). Additional analyses were done to exclude that pregnancy could be a major determinant of the difference in the prevalence of events between CD and control disease. To address this possibility, the comparison between women with CD and women with control disease was repeated after exclusion of events related to pregnancy. However, a significant difference in the prevalence of events between the CD and the control group was confirmed in this additional analysis (67.4% *vs.* 34.3%, χ^2^ 12.769, *p* < 0.001). Furthermore, 20.3% of CD women who experienced a pregnancy indicated the pregnancy itself as a stressful event, while no women who experienced a pregnancy in the control disease group did (*p* = 0.02).

Table 2 shows the distribution of life events by the 10 categories of the Paykel’s interview. The two most common categories of events in CD patients were somatic health problems and loss (prevalence > 17%). Distribution of events was similar in the Control group. CD patients reported most frequently gastrointestinal symptoms, and diagnosis of severe anemia, osteoporosis, and thyroiditis among somatic health problems.

**Table 2:** Distribution of life events among the 10 areas identified by the Paykel Scale in CD and control (GERD) subjects.

Variables	CD *n* (%)	GERD *n* (%)	*p* *
Number and percentage of patients reporting an event	125 (67.2)	36 (37.5)	<0.001
**Type of Event**			
Employment	12 (9.6)	2 (5.6)	0.6
Education	14 (11.2)	0 (0.0)	0.08
Financial status	7 (5.6)	6 (16.7)	0.07
Somatic health	39 (31.2)	10 (27.8)	0.9
Loss	22 (17.6)	6 (16.7)	0.9
Living place	7 (5.6)	2 (5.6)	0.7
Sentimental life	4 (3.2)	2 (5.6)	0.9
Criminality	1 (0.8)	0 (0.0)	0.5
Family and society	8 (6.4)	2 (5.6)	0.8
Matrimonial problems	11 (8.8)	6 (16.7)	0.3

*n* = number; * Chi square test.

Since most of somatic health problems might be considered dependent from the illness under consideration, analyses were repeated with the exclusion of the patients reporting health-related events. CD patients reported more frequently events independent from the disease when compared to the control disease group (86/147 (58.5%) *vs.* 26/86 (30.2%), χ^2^ 10.655, *p* = 0.001).

Table 3 describes the patients’ opinions about the possible relationship between the reported event and the disease development. Findings were not significantly different between the CD group and Control group (χ^2^ 1.132, *p* = 0.9) and indicated that only a minority of the patients pointed out that the event could have a role in the development of the disease.

**Table 3:** Patients’ opinion about the effect of the reported event on the development of the disease Scale in CD and control (GERD) subjects according to the Paykel scale.

Variables	CD	GERD
Number of patients reporting an event	125	36
**5-Point Rate Scale**		
No effect	52.8%	55.6%
Probably no effect	20.8%	11.1%
Uncertain	16.8%	22.2%
Probably some effect	5.6%	5.6%
Sure effect	4.0%	5.6%

Celiac patients were interviewed extensively about the life events that occurred during the year prior to the onset of symptoms. Since symptoms typically precede diagnosis by months or even years in this condition, we aimed to explore specifically whether limiting the time lapse from the diagnosis and the onset of symptoms as much as possible changes the previous findings. Then, the analyses were repeated selecting subgroups of CD and GERD patients in whom symptoms begun not more than 12 months before diagnosis. In this subgroup, CD patients reported a higher prevalence of life events than GERD patients (40/56, 71.4% *vs.* 18/48, 37.5%, χ^2^ 12.062, *p* = 0.01). This prevalence became even stronger with the exclusion of patients who experienced a pregnancy and consequently reported events (χ^2^ 8.181, *p* = 0.006).

## 4. Discussion

The main finding of the present study is that CD patients reported more frequent stressful events in the years prior to diagnosis and this result is even more significant when the analysis is limited to CD patients with onset of symptoms in the year preceding the diagnosis. In fact, also in this subgroup during the 12 months prior to CD diagnosis there is a significant higher prevalence of life events in comparison to patients with GERD, even higher than that found in the whole CD group (71.4% *vs.* 67.2%).

Reflux disease had been chosen as a control group in this study for several symptomatological similarities with CD, but for its known independence from immunological mechanisms. Moreover, it has already been demonstrated that in practice total stress scores are found to be highly correlated with number of events, since, as previously stated, most reported events are from a comparatively narrow midrange of scores [24].

Other indices related to the event—type and time before diagnosis—were similar between CD patients and GERD patients, although severity of the events, assessed by the normative values was higher in GERD patients than CD. Somatic health problems and death of a close relative were the most frequent events. CD patients complain of some other co-morbidities, such as thyroid diseases and diabetes, that could obscure the association between life events and the onset of disease. With the exclusion of patients reporting those health problems as “stressful events”, the number of stressful events in celiac disease was still greater than that reported in the GERD patients. Among CD patients, the frequency of stressful events prior to diagnosis was significantly higher in women than men. Analyzing the type of events, we noticed that pregnancy was reported as a stressful event in (20.5%) cases by celiac women but never by women in the control group. This could at least in part explain the higher percentage of stressful events reported by CD women. Pregnancy is a peculiar moment in the fertile life of an undiagnosed celiac woman [28] also because pregnancy may disclose the relative “subclinical” deficiency of iron or other nutrients by increasing the metabolic demand and also may favor the development of the so-called “celiac crisis” that is an acute phase of open malabsorption leading to gluten-related auto-antibodies search [29]. One cannot exclude, however, that during pregnancy a combination of somatic and psychological factors may equally contribute to the request for medical help by the celiac women who are not yet aware of their disease. In the present study, pregnancy was one the events which could be selected by the patient in the Paykel’s interview. It is possible that celiac women could have perceived their pregnancy as a negative event more frequently than women with the control disease because of the metabolic imbalance associated with malabsorption. To address this possibility, the comparison between CD women and GERD group women was repeated after exclusion of events related to pregnancy and our results demonstrated that celiac women still remained more sensitive to psychosocial stressors.

As symptoms due to gluten intolerance may precede by years the diagnosis of celiac disease, a separate analysis of data from a subgroup of subjects in whom the diagnosis was made within one year from the onset of symptoms was planned and the results fully confirmed the above findings.

The interpretation of the results of this study is complex because the available evidence indicates that the relationship between life stress events and the course of a disease is likely dependent on various factors. Little is known about how these factors, individually or in combination, are related to any disease activity. The nature and strength of these inter-relationships have strong clinical implications not for a single disease but for understanding the pathogenesis and the course of several pathologies. Patients with a long-lasting, obscure, oscillating illness experience various stressful events, which affect life patterns and give rise to conflicts. So the patients might become entangled in a vicious circle where life events accelerate the appearance of a disease or its symptoms which, in turn, facilitates the occurrence of life events, as for instance the loss of work or the onset of economic problems. It is well known that CD patients often experience altered psychological behavior during their life [30,31,32]. Other, alternative possibilities are that an event could push a person to a medical consultation because pre-existing symptoms become more important in his/her perception or that the event affected the primary immunological mechanism of the disease. The first possibility however is not in accordance with our findings of this subgroup of celiac patients in whom diagnosis was made close to the onset of symptoms who reported a greater number of life events compared to controls.

Moreover, in the case of CD, we know that CD may run undiagnosed for years and that subtle symptoms may be underestimated by patients until after treatment. In those cases the evaluation of the precise time of onset of symptoms and effect of stressful events on the diagnosis the might be hard to estimate.

Psychological stress has been repeatedly reported to increase disease activity in gastrointestinal diseases [33] and recent studies have confirmed that adverse life events, chronic stress, and depression increase the likelihood of relapse in patients with quiescent IBD seemingly through changes in hypothalamic-pituitary-adrenal axis function, alterations in bacterial-mucosal interactions, mucosal mast cells and mediators such as corticotrophin releasing factor [7,34]. These observations were not confirmed by a population-based studies focused on the role of life events in determining the onset of inflammatory bowel diseases [35,36].

There are several limitations in our study. First of all, the lack of sample size calculation that might lead to a misinterpretation of results. This factor could be less of a concern in the majority of results since significant differences were reported. Another point is that our sample group might not represent all CD patients since they were studied in a tertiary care center. Despite the possibility through the Paykel’s scale to point out major and minor health problems, for the lack of information about the precise diagnosis we were unable to assess if those events were related to the pathogenesis of CD, as suggested in previous studies [37,38]. Lastly, the recall bias as pointed out in the introduction, although we tried to reduce it limiting the time lapse from the onset of symptoms and the diagnosis to one year in all CD and GERD patients.

## 5. Conclusions

In conclusion, our study indicates that life-events are associated to some degree with recent diagnosis of celiac disease in adults. The number of the events and not their severity appear as the determinant factor. Our data indicate that stressful events preceding celiac disease diagnosis are particularly frequent among celiac women, including pregnancy, which is defined as a stressful event only by celiac women and not by control women with gastroesophageal reflux. Altogether, also the present data support the need for psychological support in celiac disease, particularly in women at the time of celiac disease diagnosis [39,40].

## References

[1] Addolorato G., Capristo E., Ghittoni G., Valeri C., Mascianà R., Ancona C., Gasbarrini G. (2001). Anxiety but not depression decreases in coeliac patients after one-year gluten-free diet: A longitudinal study. Scand. J. Gastroenterol..

[2] Hallert C., Grännö C., Hultén S., Midhagen G., Ström M., Svensson H., Valdimarsson T. (2002). Living with coeliac disease: Controlled study of the burden of illness. Scand. J. Gastroenterol..

[3] Renzaho A.M., Houng B., Oldroyd J., Nicholson J.M., D’Esposito F., Oldenburg B. (2013). Stressful life events and the onset of chronic diseases among australian adults: Findings from a longitudinal survey. Eur. J. Public Health.

[4] Vidal A., Gomez-Gil E., Sans M., Portella M.J., Salamero M., Pique J.M., Panes J. (2006). Life events and inflammatory bowel disease relapse: A prospective study of patients enrolled in remission. Am. J. Gastroenterol..

[5] Wolters F.L., Russel M.G., Sijbrandij J., Schouten L.J., Odes S., Riis L., Munkholm P., Langholz E., Bodini P., O’Morain C. (2006). Disease outcome of inflammatory bowel disease patients: General outline of a Europe-wide population-based 10-year clinical follow-up study. Scand. J. Gastroenterol. Suppl..

[6] Maunder R.G., Levenstein S. (2008). The role of stress in the development and clinical course of inflammatory bowel disease: Epidemiological evidence. Curr. Mol. Med..

[7] Mawdsley J.E., Rampton D.S. (2005). Psychological stress in ibd: New insights into pathogenic and therapeutic implications. Gut.

[8] Brown R.F., Tennant C.C., Sharrock M., Hodgkinson S., Dunn S.M., Pollard J.D. (2006). Relationship between stress and relapse in multiple sclerosis: Part II. Direct and indirect relationships. Mult. Scler..

[9] Geenen R., van Middendorp H., Bijlsma J.W. (2006). The impact of stressors on health status and hypothalamic-pituitary-adrenal axis and autonomic nervous system responsiveness in rheumatoid arthritis. Ann. NY. Acad. Sci..

[10] Beveridge R.M., Berg C.A., Wiebe D.J., Palmer L.D. (2006). Mother and adolescent representations of illness ownership and stressful events surrounding diabetes. J. Pediatr. Psychol..

[11] Picardi A., Abeni D. (2001). Stressful life events and skin diseases: Disentangling evidence from myth. Psychother. Psychosom..

[12] Kennedy S., Thompson R., Stancer H.C., Roy A., Persad E. (1983). Life events precipitating mania. Br. J. Psychiatry.

[13] Hammen C., Gitlin M. (1997). Stress reactivity in bipolar patients and its relation to prior history of disorder. Am. J. Psychiatry.

[14] Johnson L., Andersson-Lundman G., Aberg-Wistedt A., Mathe A.A. (2000). Age of onset in affective disorder: Its correlation with hereditary and psychosocial factors. J. Affect. Disord..

[15] Rampton D.S. (2011). The influence of stress on the development and severity of immune-mediated diseases. J. Rheumatol. Suppl..

[16] Iovino P., Pascariello A., Russo I., Galloro G., Pellegrini L., Ciacci C. (2013). Difficult diagnosis of celiac disease: Diagnostic accuracy and utility of chromo-zoom endoscopy. Gastrointest. Endosc..

[17] Marsh M.N. (1992). Gluten, major histocompatibility complex, and the small intestine. A molecular and immunobiologic approach to the spectrum of gluten sensitivity (“celiac sprue”). Gastroenterology.

[18] Ciacci C., Iavarone A., Mazzacca G., de Rosa A. (1998). Depressive symptoms in adult coeliac disease. Scand. J. Gastroenterol..

[19] Zung W.W. (1973). From art to science. The diagnosis and treatment of depression. Arch. Gen. Psychiatry.

[20] Green P.H. (2005). The many faces of celiac disease: Clinical presentation of celiac disease in the adult population. Gastroenterology.

[21] Amato G., Limongelli P., Pascariello A., Rossetti G., del Genio G., del Genio A., Iovino P. (2008). Association between persistent symptoms and long-term quality of life after laparoscopic total fundoplication. Am. J. Surg..

[22] Santonicola A., Siniscalchi M., Capone P., Gallotta S., Ciacci C., Iovino P. (2012). Prevalence of functional dyspepsia and its subgroups in patients with eating disorders. World J. Gastroenterol..

[23] Paykel E.S. (1983). Methodological aspects of life events research. J. Psychosom. Res..

[24] Paykel E.S. (1997). The interview for recent life events. Psychol. Med..

[25] Paykel E.S. (2001). The evolution of life events research in psychiatry. J. Affect. Disord..

[26] Canestrari R. (1982). Versione Italiana Della Scala di Paykel per Gli Eventi Stressanti. Nuovi Metodi in Psicometria.

[27] (2006). SPSS 15.0 Command Syntax Reference 2006.

[28] Santonicola A., Iovino P., Cappello C., Capone P., Andreozzi P., Ciacci C. (2011). From menarche to menopause: The fertile life span of celiac women. Menopause.

[29] Smecuol E., Maurino E., Vazquez H., Pedreira S., Niveloni S., Mazure R., Boerr L., Bai J.C. (1996). Gynaecological and obstetric disorders in coeliac disease: Frequent clinical onset during pregnancy or the puerperium. Eur. J. Gastroenterol. Hepatol..

[30] Ciacci C., Iavarone A., Siniscalchi M., Romano R., de Rosa A. (2002). Psychological dimensions of celiac disease: Toward an integrated approach. Dig. Dis. Sci..

[31] De Rosa A., Troncone A., Vacca M., Ciacci C. (2004). Characteristics and quality of illness behavior in celiac disease. Psychosomatics.

[32] Ciacci C., de Rosa A., de Michele G., Savino G., Squillante A., Iovino P., Sabbatini F., Mazzacca G. (1998). Sexual behaviour in untreated and treated coeliac patients. Eur. J. Gastroenterol. Hepatol..

[33] Stanghellini V. (1999). Relationship between upper gastrointestinal symptoms and lifestyle, psychosocial factors and comorbidity in the general population: Results from the domestic/international gastroenterology surveillance study (digest). Scand. J. Gastroenterol. Suppl..

[34] Mawdsley J.E., Rampton D.S. (2006). The role of psychological stress in inflammatory bowel disease. Neuroimmunomodulation.

[35] Fritze J., Schneider B., Maurer K. (1996). Additive effects, but no synergistic interaction of stressful life-events and genetic loading in affective disorders. J. Neural Transm..

[36] Lerebours E., Gower-Rousseau C., Merle V., Brazier F., Debeugny S., Marti R., Salomez J.L., Hellot M.F., Dupas J.L., Colombel J.F. (2007). Stressful life events as a risk factor for inflammatory bowel disease onset: A population-based case-control study. Am. J. Gastroenterol..

[37] Riddle M.S., Murray J.A., Porter C.K. (2012). The incidence and risk of celiac disease in a healthy US adult population. Am. J. Gastroenterol..

[38] Maple J.T., Pearson R.K., Murray J.A., Kelly D.G., Lara L.F., Fan A.C. (2007). Silent celiac disease activated by pancreaticoduodenectomy. Dig. Dis. Sci..

[39] Addolorato G., de Lorenzi G., Abenavoli L., Leggio L., Capristo E., Gasbarrini G. (2004). Psychological support counselling improves gluten-free diet compliance in coeliac patients with affective disorders. Aliment. Pharmacol. Ther..

[40] Siniscalchi M., Iovino P., Tortora R., Forestiero S., Somma A., Capuano L., Franzese M.D., Sabbatini F., Ciacci C. (2005). Fatigue in adult coeliac disease. Aliment. Pharmacol. Ther..

